# L-lactic acid production from D-xylose with *Candida sonorensis* expressing a heterologous lactate dehydrogenase encoding gene

**DOI:** 10.1186/s12934-014-0107-2

**Published:** 2014-08-08

**Authors:** Kari T Koivuranta, Marja Ilmén, Marilyn G Wiebe, Laura Ruohonen, Pirkko Suominen, Merja Penttilä

**Affiliations:** VTT Technical Research Centre of Finland, P. O. Box 1000, Espoo, FI-02044 VTT Finland; Cargill Biotechnology Research and Development, 15285 Minnetonka Blvd, Minnetonka, MN 55345 USA

**Keywords:** *Candida sonorensis*, Yeast, D-xylose, L-lactic acid production, Xylose isomerase, Pyruvate decarboxylase, Xylose reductase, Xylitol dehydrogenase

## Abstract

**Background:**

Bioplastics, like polylactic acid (PLA), are renewable alternatives for petroleum-based plastics. Lactic acid, the monomer of PLA, has traditionally been produced biotechnologically with bacteria. With genetic engineering, yeast have the potential to replace bacteria in biotechnological lactic acid production, with the benefits of being acid tolerant and having simple nutritional requirements. Lactate dehydrogenase genes have been introduced to various yeast to demonstrate this potential. Importantly, an industrial lactic acid producing process utilising yeast has already been implemented. Utilisation of D-xylose in addition to D-glucose in production of biochemicals such as lactic acid by microbial fermentation would be beneficial, as it would allow lignocellulosic raw materials to be utilised in the production processes.

**Results:**

The yeast *Candida sonorensis*, which naturally metabolises D-xylose, was genetically modified to produce L-lactic acid from D-xylose by integrating the gene encoding L-lactic acid dehydrogenase (*ldhL*) from *Lactobacillus helveticus* into its genome. In microaerobic, CaCO_3_-buffered conditions a *C. sonorensis ldhL* transformant having two copies of the *ldhL* gene produced 31 g l^−1^ lactic acid from 50 g l^−1^ D-xylose free of ethanol.

Anaerobic production of lactic acid from D-xylose was assessed after introducing an alternative pathway of D-xylose metabolism, i.e. by adding a xylose isomerase encoded by *XYLA* from *Piromyces sp*. alone or together with the xylulokinase encoding gene *XKS1* from *Saccharomyces cerevisiae*. Strains were further modified by deletion of the endogenous xylose reductase encoding gene, alone or together with the xylitol dehydrogenase encoding gene. Strains of *C. sonorensis* expressing xylose isomerase produced L-lactic acid from D-xylose in anaerobic conditions. The highest anaerobic L-lactic acid production (8.5 g l^−1^) was observed in strains in which both the xylose reductase and xylitol dehydrogenase encoding genes had been deleted and the xylulokinase encoding gene from *S. cerevisiae* was overexpressed.

**Conclusions:**

Integration of two copies of the *ldhL* gene in *C. sonorensis* was sufficient to obtain good L-lactic acid production from D-xylose. Under anaerobic conditions, the *ldhL* strain with exogenous xylose isomerase and xylulokinase genes expressed and the endogenous xylose reductase and xylitol dehydrogenase genes deleted had the highest L- lactic acid production.

## Background

Lactic acid and its derivatives, e.g. biodegradable polymers, are widely used in food, chemical, cosmetic and pharmaceutical industries [1]. Today, L-lactic acid is commercially manufactured predominantly by bacterial fermentation. However, the bacterial process has both nutritional (complex medium) and pH (neutral pH) requirements which increase the cost of the process [1,2]. To improve the economics of the process, use of yeast for L-lactic acid production instead of bacteria has already been implemented by Cargill Inc [3]. Producing L-lactic acid with yeast enables the use of cheaper growth media and low pH cultures, the latter allowing down-stream recovery of L-lactic acid without producing as much gypsum waste as at high pH [2,4]. Production of L-lactic acid from D-glucose has been described with several yeast species, e.g. *Saccharomyces cerevisiae, Kluyveromyces lactis, Scheffersomyces stipitis* and *Candida sonorensis,* expressing L-lactate dehydrogenase encoding genes originating from different organisms [4-6].

Cheap growth media could include sugars derived from hydrolysed plant biomass which contains in addition to hexoses (e.g. D-glucose), significant amounts of pentoses (e.g. D-xylose) [7]. To achieve cost-effective bioprocesses based on plant biomass both hexoses and pentoses need to be consumed and converted to the desired product by the process organism. Production of L-lactic acid from D-xylose or D-xylose-containing raw materials has been shown with several bacterial species [5]. In eukaryotes, D-xylose-derived L-lactic acid production has been reported with the filamentous fungus *Rhizopus oryzae* [8-10] and with the genetically modified yeast species *S. stipitis* and *Candida utilis* expressing the *LDH* genes of *Lactobacillus helveticus* and *Bos taurus*, respectively [11,12].

D-xylose can be converted to D-xylulose, and further to pyruvate, via the oxidoreductive xylose reductase – xylitol dehydrogenase pathway. D-Xylose is first reduced to xylitol with a NAD(P)H-dependent xylose reductase (XR encoded by *XYL1*), and xylitol further oxidised to D-xylulose with a NAD^+^-dependent xylitol dehydrogenase (XDH encoded by *XYL2*) [13]. *S. stipitis* like *Pachysolen tannophilus* and *Candida shehatae* can convert D-xylose to ethanol under aerobic or oxygen-limited conditions [14]. The ability of these three yeast species to ferment D-xylose under oxygen-limited conditions most likely reflects the fact that they each possess a xylose reductase with dual cofactor specificity (NADH and NADPH), whereas fungal species incapable of D-xylose fermentation have strictly NADPH-dependent xylose reductases. Without the dual cofactor specificity of xylose reductase, the overall redox neutral conversion of D-xylose to D-xylulose by xylose reductase and xylitol dehydrogenase results in a redox cofactor imbalance [13,14]. The xylose reductase and xylitol dehydrogenase encoding genes from *S. stipitis* have been expressed in *S. cerevisiae*, which lacks a functional endogenous D-xylose pathway, to construct D-xylose-fermenting, ethanol-producing strains [13,14].

The D-xylose utilisation pathway common in bacteria involves xylose isomerase, which directly isomerises D-xylose to D-xylulose without any cofactors. Thus, a strategy for avoiding redox imbalance during D-xylose metabolism in yeast is the introduction of a xylose isomerase encoding gene of bacterial [15-18] or fungal [19,20] origin. However, only some xylose isomerases have been shown to be functional in yeast; e.g. the bacterial xylose isomerases of *Thermus thermophilus* [21], *Escherichia coli* [16,22], *Streptomyces coelicolor* [16], *Clostridium phytofermentans* [17] and *Bacteroides stercoris* [18] and most importantly, the eukaryotic xylose isomerases from the anaerobic cellulolytic fungi *Piromyces* sp E2 [19] and *Orpinomyces* [20].

Bacterial genes encoding a xylose isomerase have also been expressed in yeast species which have an endogenous pathway of D-xylose metabolism to enhance ethanol production. Overexpression of the *E. coli* xylose isomerase encoding gene in *Schizosaccharomyces pombe* resulted in ethanol production, which was not seen with the wild type strain [22]. Overexpression of *E. coli* or *S. coelicolor* xylose isomerase encoding genes in a *Hansenula polymorpha* strain from which the xylose reductase encoding gene had been deleted resulted in ethanol production comparable to the native *H. polymorpha* host, even though the xylose isomerase activity was only 20% of that present in the bacterial host strains [16].

In the present study we used a *Candida sonorensis* strain expressing the *L. helveticus ldhL* gene to assess L-lactic acid production from D-xylose. This yeast has been shown to produce L-lactic acid from D-glucose at low pH [6]. In addition to lactic acid production with the endogenous xylose reductase - xylitol dehydrogenase pathway we demonstrate for the first time lactic acid production via the xylose isomerase pathway in yeast. We present data on enhanced anaerobic L-lactic acid production by a *C. sonorensis ldhL* strain by the expression of *Piromyces XYLA* alone or with *S. cerevisiae XKS1* in strains (i) with an intact endogenous D-xylose pathway and (ii) in which the xylose reductase encoding gene alone or in combination with the xylitol dehydrogenase encoding gene has been deleted.

## Results

### L-lactic acid production from D-xylose by a *Candida sonorensis ldhL* strain

To assess L-lactic acid production from D-xylose the wild type and transformant C29, with one copy of *ldhL*, were cultivated in buffered D-xylose (50 g l^−1^) medium (YXC) in microaerobic conditions. During the 168 h cultivation, the biomass of the C29 transformant and wild type increased from OD_600_ 10 to 26 (±1.7) and 19 (±2.2), respectively. C29 consumed all D-xylose provided, whereas the wild type strain did not; 9.2 ± 2.9 g residual D-xylose l^−1^ was observed at the end of the cultivation with the wild type. No L-lactic acid was produced by the wild type strain, which produced 7.8 ± 1.1 g xylitol l^−1^, 4.0 ± 0.9 g ethanol l^−1^ and 6.5 ± 0.9 g acetate l^−1^ (Table 1). The C29 transformant produced 26.7 ± 0.8 g L-lactic acid l^−1^ with a yield of 0.53 ± 0.03 (g L-lactic acid per g D-xylose consumed). Neither ethanol nor acetate was produced by C29, but some D-xylose was reduced to xylitol (4.6 ± 0.5 g l^−1^), all of which was consumed before the end of the cultivation.

**Table 1:** **Maximum L-lactic acid and xylitol production in microaerobic cultivation**

**Strain**	**Genotype**	**L-lactic acid (g l** ^**−1**^ **)**	**L-lactic acid yield (g g** ^**−1**^ **)**	**Xylitol (g l** ^**−1**^ **)**
Wild type^1^		n.d.	n.d.	7.8 ± 1.1*
C29	*x::ldhL*	26.7 ± 0.8	0.53 ± 0.03	4.6 ± 0.5
C184	*pdc1Δ::ldhL pdc2Δ*	27.3 ± 0.8	0.53 ± 0.02	3.9 ± 0.7
C169	*pdc1Δ::ldhL pdc2Δ::ldhL*	30.8 ± 1.0*	0.57 ± 0.03	5.6 ± 0.5

^1^The wild type strain also produced 4.0 ± 0.9 g ethanol l^−1^ and 6.5 ± 0.9 g acetate l^−1^.

Maximum concentrations of L-lactic acid and xylitol produced from D-xylose by *C. sonorensis* wild type and C29, C169 and C184, and yield of L-lactic acid from D-xylose. Cells were cultivated in buffered YNB medium with 50 g l^−1^ D-xylose medium at 30°C, 100 rpm. Data are mean ± sem from 3 to 12 replicate cultures. n.d. = not detected. An asterisk indicates that the value is significantly different (p < 0.05) from other values in the same column.

### Pre-cultivation on D-xylose improved L-lactic acid production by the *Candida sonorensis ldhL* strain

**Figure 1:**
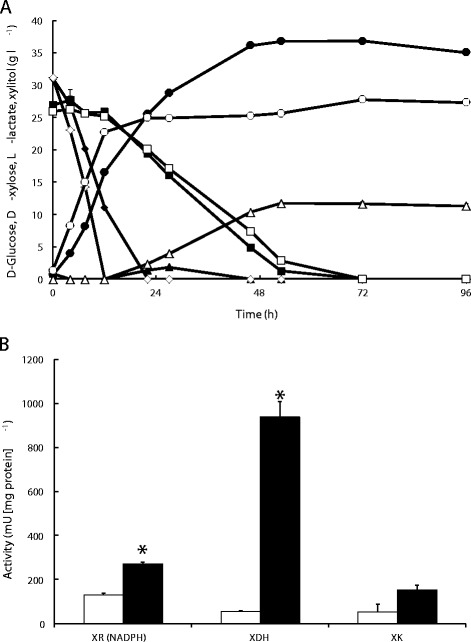
**D-Glucose and D-xylose consumption and xylitol and L-lactic acid production in buffered microaerobic cultivation. (A)**. Consumption of D-glucose [♦◊] and D-xylose [■□], and production of xylitol [▲Δ] and lactic acid [●○] by *C. sonorensis* C169 (2*ldhL, *pdc1Δ*, *pdc2Δ*) in CaCO_3_-buffered YNB with 30 g l^−1^ D-glucose and 30 g l^−1^ D-xylose at 30°C, 100 rpm. Cultures were inoculated with cells which had been pre-cultured on D-xylose (closed symbols) or D-glucose (open symbols). **(B)**. Xylose reductase (XR, NADPH-dependent), xylitol dehydrogenase (XDH) and xylulokinase (XK) activities assayed after 27 h of cultivation in CaCO_3_-buffered YNB with 30 g l^−1^ D-glucose and 30 g l^−1^ D-xylose. Cultures were inoculated with D-xylose (black bars) or D-glucose (white bars) grown cells. Results are averages ± sem from duplicate flask cultivations. Where error bars are not visible they were smaller than the symbol. An asterisk indicates that the value is significantly different (p < 0.05) from that of the cultures inoculated with D-glucose-grown cells.

In the preliminary cultivations the biomass of *ldhL* transformants was generated either on D-glucose or D-xylose. After transfer of the biomass to buffered D-xylose (50 g l^−1^) medium (YXC), lower production of L-lactic acid was observed in cultures inoculated with D-glucose-grown cells than in cultures inoculated with D-xylose-grown cells (data not shown). In addition, when C169 (*PDC-,* 2*ldhL; see below) was pre-cultured on either D-glucose (YGM) or D-xylose (YXM) to obtain biomass for a cultivation in buffered D-glucose (30 g l^−1^) and D-xylose (30 g l^−1^) medium (YGXC) (Figure 1), the cultures inoculated with D-glucose-grown cells consumed D-glucose faster (2.6 g l^−1^ h^−1^; p <0.05) than the culture inoculated with D-xylose-grown cells (1.7 g l^−1^ h^−1^), while consuming D-xylose more slowly (0.54 ± 0.01 and 0.60 ± 0.00 g l^−1^ h^−1^, for cultures inoculated with D-glucose- and D-xylose-grown cells, respectively).

Cultures inoculated with D-xylose-grown cells produced more L-lactic acid (37 ± 0.2 g l^−1^; p <0.05) and less xylitol (1.9 ± 0.1 g l^−1^) than cultures inoculated with D-glucose-grown cells (28 ± 0.1 g l^−1^ L-lactic acid and 11.6 ± 0.2 g l^−1^ xylitol). Most of the L-lactic acid was produced from D-glucose when cultures were inoculated with D-glucose-grown cells, whereas approximately 12 g l^−1^ L-lactic acid (out of the total 37 g l^−1^) was produced from D-xylose after D-glucose had been consumed in cultures inoculated with D-xylose-grown cells (Figure 1A). Additionally, the xylitol produced was subsequently consumed in the cultures inoculated with D-xylose-grown cells, but not in the cultures inoculated with D-glucose-grown cells.

### Pre-cultivation on D-xylose increased the activities of xylose reductase, xylitol dehydrogenase and xylulokinase as well as mRNA levels of the corresponding genes in the *C. sonorensis ldhL* strain

Xylose reductase, xylitol dehydrogenase and xylulokinase activities were assayed from the C169 cells after 27 h of cultivation on D-xylose, after inoculation with either D-glucose- or D-xylose-grown cells. The NADPH-dependent xylose reductase activity per mg protein was 130 and 270 mU in cultures inoculated with D-glucose- and D-xylose-grown cells, respectively (Figure 1B). No NADH-dependent xylose reductase activity was detected. The xylitol dehydrogenase activities were 60 and 940 mU/mg protein and xylulokinase activities 50 and 150 mU/mg protein in cultures inoculated with D-glucose- or D-xylose-grown precultures, respectively.

**Figure 2:**
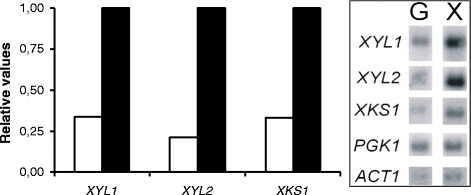
**Northern analysis of mRNA levels of** ***XYL1*** **,** ***XYL2*** **and** ***XKS1*** **in** ***C*** **.** ***sonorensis*** **C169.** RNA was isolated from cultures grown for 20 h on YNB medium containing 50 g l^−1^ D-glucose (G, white bars) or D-xylose (X, black bars). The left panel shows relative gene expression, with the expression level in cells growing on D-xylose = 1 of the hybridisation signals shown in the right panel. *C. sonorensis XYL1*, *XYL2*, *XKS1* and *PGK1* and *S. cerevisiae ACT1* genes were used as probes. The hybridisation signals were normalized to total RNA.

A Northern analysis was carried out to analyse the mRNA levels of genes encoding xylose reductase (*XYL1*), xylitol dehydrogenase (*XYL2*) and xylulokinase (*XKS1*) in the D-glucose- and D-xylose-grown C169 cells. The *XYL1, XYL2* and *XKS1* genes had 3-, 5- and 3-fold higher mRNA levels in cells grown on D-xylose compared to cells grown on D-glucose (Figure 2). Comparable differences in the mRNA levels of *XYL1, XYL2* and *XKS1* in D-glucose- and D-xylose-grown cells were seen also in the wild type *C. sonorensis* strain (data not shown).

### Effect of *PDC* deletion and copy number of the *ldhL* gene on L-lactic acid production

Several *C. sonorensis* transformants with one to three copies of *ldhL* integrated in the genome, and one or both of the *PDC* genes deleted were previously constructed and characterised for L-lactic acid production on D-glucose [6]. To analyse the effect of deletion of both *PDC1* and *PDC2* (C184) on L-lactic acid production from D-xylose, strains with one copy of *ldhL,* with (C29) or without (C184) intact *PDC* genes, were cultivated in buffered D-xylose (50 g l^−1^) medium (YXC) in microaerobic conditions (Table 1). Both strains produced 27 g L-lactic acid l^−1^ during the 168 hours cultivation. Additionally, C29 and C184 produced similar amounts of xylitol (4.6 ± 0.5 g l^−1^ and 3.9 ± 0.7 g l^−1^, respectively) during the cultivation, and both strains consumed all the xylitol by the end of the cultivation. Both strains produced similar amounts of biomass, and neither of the strains produced ethanol or acetate under the conditions studied.

To test the effect of the number of copies of the *ldhL* gene on L-lactic acid production from D-xylose, C169 was cultivated in the same conditions as C29 and C184 (Table 1). This *pdc1-* strain, with two copies of *ldhL*, produced slightly more L-lactic acid (30.8 ± 1.0 g l^−1^; p < 0.05) than the single copy strains (C29 and C184, Table 1). C169 also produced slightly more xylitol (5.6 ± 0.5 g l^−1^; t-test p < 0.05, ANOVA p > 0.05) than C184. Xylitol was consumed by both strains by the end of the cultivation. C169 produced less biomass (OD_600_ 22) than C184 (OD_600_ 26).

In non-buffered conditions, i.e. lacking CaCO_3_, *C. sonorensis* C169 (2 copies of *ldhL*) and C184 (1 copy *ldhL*) both produced approximately 13 g l^−1^ L-lactic acid from 24 g l^−1^ D-xylose (yield ~0.5 g g^−1^). L-Lactic acid production and D-xylose consumption stopped after 41 hours when the pH had decreased from 5.4 to 2.8 (data not shown).

### L-lactic acid production from D-xylose by a *C. sonorensis ldhL* strain expressing *Piromyces sp. XYLA* and *S. cerevisiae XKS1* genes

To verify the functional expression of the *Piromyces sp.* E2 *XYLA* gene encoding xylose isomerase (*XYLA*) in *C. sonorensis, XYLA* was expressed alone or together with the *S. cerevisiae XKS1* gene encoding xylulokinase (*ScXKS1*) in a *C. sonorensis* strain carrying one copy of the *ldhL* gene. Strains with *ldhL* (C29), *ldhL* with *XYLA* (C281) or *ldhL* with *XYLA* and *ScXKS1* (C282-C284) were cultivated in CaCO_3_-buffered YP medium with 50 g l^−1^ D-xylose. Xylose isomerase activity (100 mU mg^−1^) was measurable after 24 h cultivation, indicating that the *XYLA* encoded enzyme was functionally expressed in *C. sonorensis* (Table 2). C281 and C282-C284 consumed D-xylose at a higher (p < 0.05) rate (0.48 ± 0.01 g D-xylose l^−1^ h^−1^) than C29 (0.43 ± 0.00 g D-xylose l^−1^ h^−1^) and produced slightly more biomass. C282-C284 produced less xylitol (6.7 ± 0.2 g l^−1^) than C29 (8.1 ± 0.3 g l^−1^) or C281 (8.7 ± 0.2 g l^−1^; Table 3). Xylitol was consumed before the end of the cultivation. These strains produced similar amounts of L-lactic acid (31 g l^−1^) at similar rates (0.27 ± 0.01 g l^−1^ h^−1^ for C29, 0.26 ± 0.01 g l^−1^ h^−1^ for C281 and 0.29 ± 0.00 g l^−1^ h^−1^ for C282-C284). The strain with only *XYLA* (C281) accumulated more D-xylulose (0.63 g l^−1^) than either the control (C29, 0.43 g D-xylulose l^−1^) or C282-C284 with both *XYLA* and *ScXKS1* (0.45 g D-xylulose l^−1^).

**Table 2:** **Xylose isomerase activity**

**Strain**	**Genotype**	**XI activity (mU mg** ^**−1**^ **)**
C29	*ldhL*	10 ± 0
C281	*ldhL, XYLA*	110 ± 10
C282-C284	*ldhL, XYLA, ScXKS1*	90 ± 10

Xylose isomerase (XI) activity in *C. sonorensis* C29 containing *ldhL,* and in *ldhL* strains expressing the *Piromyces sp. XYLA* gene alone (C281) or with the *S. cerevisiae XKS1* gene (C282-C284). The activity was measured from cell free extracts prepared after overnight cultivation of cells in YP with 50 g l^−1^ D-glucose and 10 mM MgCl_2_. The activity values are means ± sem from 3 (C29, C281) or 6 (C282-C284, duplicates of three independent transformants) aerobic flask cultivations.

**Table 3:** **L-lactic acid, xylitol and D-xylulose production with** ***XYLA*** **strains in microaerobic cultivation**

**Strains**	**L-lactic acid (g l** ^**−1**^ **)**	**L-lactic acid yield (g g** ^**−1**^ **)**	**Xylitol (g l** ^**−1**^ **)**	**D-Xylulose (g l** ^**−1**^ **)**
C29	31.0 ± 0.3	0.59 ± 0.01	8.1 ± 0.3*	0.43 ± 0.01
C281	30.9 ± 0.3	0.60 ± 0.01	8.7 ± 0.2*	0.63 ± 0.02*
C282-C284	31.8 ± 0.1*	0.61 ± 0.00	6.7 ± 0.2	0.45 ± 0.00

Concentrations (g l^−1^) of L-lactic acid, xylitol, and D-xylulose produced and yield of L-lactic acid on D-xylose consumed (g g^−1^) by *C. sonorensis ldhL* strains in which the native D-xylose pathway was intact (C29, C281 and C282-C284). All strains except C29 expressed *XYLA* alone (C281) or with *ScXKS1* (C282-C284)*.* Cells were cultivated in CaCO_3_ buffered, YP medium with 50 g l^−1^ D-xylose and 10 mM MgCl_2_ at 30°C, 100 rpm. Results are the mean ± sem from 3 to 6 replicate flasks. An asterisk indicates that the value is significantly different (p < 0.05) from values in the same column without an asterisk.

### Identification of xylose reductase and xylitol dehydrogenase encoding genes from *C. sonorensis*

In order to study the functionality of the XYLA pathway in the absence of the endogenous oxido-reductive D-xylose pathway the *XYL1* and *XYL2* genes were cloned and deleted.

The xylose reductase homologue *XYL1* isolated from the genomic library of *C. sonorensis* contains a protein coding region of 960 bp. The predicted amino acid sequence of *XYL1* (319 aa) had 76% overall identity with known fungal xylose reductases (data not shown). Southern analysis at low stringency detected only the *XYL1* gene. *XYL1* was confirmed to code for a xylose reductase in *XYL1* deletion strains (C689 and C690), which did not grow on D-xylose (data not shown) and had no detectable xylose reductase activity (Table 4).

**Table 4:** **Xylose reductase and xylitol dehydrogenase activities in** ***xyl1Δ*** **and** ***xyl2Δ*** **strains**

**Strains**	**Genotype**	**Xylose reductase activity**	**Xylitol dehydrogenase activity**
		**mU [mg protein]** ^**−1**^	**mU [mg protein]** ^**−1**^
wild type		75 ± 1.6	571 ± 21
C689, C690	*xyl1Δ*	2 ± 0.3	625 ± 47
C684, C685, C686	*xyl2Δ*	74 ± 0.9	126 ± 4

NADPH-dependent xylose reductase and xylitol dehydrogenase activities in *C. sonorensis* strains in which the *XYL1* or *XYL2* gene were disrupted. Wild type *C. sonorensis*, *xyl1Δ* strains (C689 and C690) and *xyl2Δ* strains (C684, C685 and C686) were grown in YNB with 50 g l^−1^ D-xylose for 24 h. Results are mean ± sem for duplicate activity measurements of wild type strain, two *xyl1Δ* strains or three *xyl2Δ* strains.

The xylitol dehydrogenase homologue *XYL2* contains a 1065 bp protein coding region. The deduced amino acid sequence had at most 74% identity with known fungal xylitol dehydrogenases (data not shown). Southern analysis at low stringency detected some other weakly hybridising genomic fragments in addition to *XYL2. XYL2* was characterised as a xylitol dehydrogenase encoding gene by deleting it from the wild type *C. sonorensis* strain. The *XYL2* deletion strains (C684, C685 and C686) grew slower on D-xylose (data not shown) and had only ~20% of xylitol dehydrogenase activity compared to the wild type (126 ± 4 mU/mg and 571 ± 21 mU/mg, respectively) (Table 4).

### L-lactic acid production in anaerobic conditions by *C. sonorensis* expressing *XYLA* and *ScXKS1* but lacking the native D-xylose pathway

**Figure 3:**
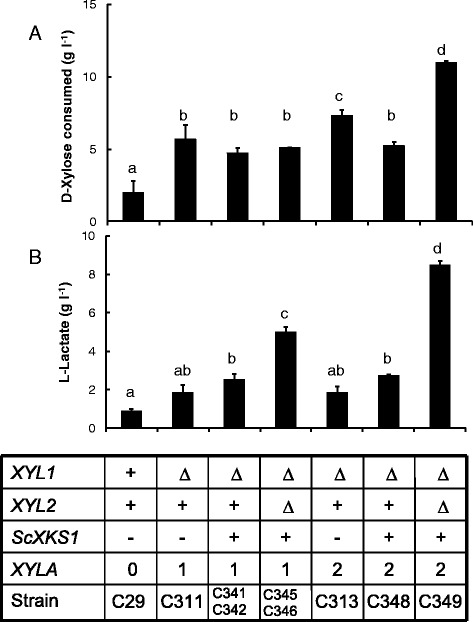
**Xylose consumption and L-lactic acid production in anaerobic cultivation.** Concentration (g l^−1^) of D-xylose consumed **(A)** and L-lactic acid produced **(B)** in anaerobic cultivations of *C. sonorensis ldhL* strains with the native D-xylose pathway disrupted and expressing *XYLA* alone (C311, *XYLA*, *xyl1*Δ; C313, 2*XYLA, *xyl1*Δ) or together with *ScXKS1* (C341-C342, *XYLA*, *ScXKS1*, *xyl1*Δ; C345-C346, *XYLA*, *ScXKS1*, *xyl1*Δ, *xyl2*Δ; C348, 2*XYLA, *ScXKS1*, *xyl1*Δ; C349, 2*XYLA, *ScXKS1*, *xyl1*Δ, *xyl2*Δ), with C29 as the control. Strains were cultivated at 30°C in 50 ml of CaCO_3_-buffered, YP-50 g l^−1^ D-xylose with 10 mM MgCl_2_ in 100 ml flasks sealed with water locks for 146 h. Initial OD600 was 12. Results are averages of duplicate flasks. Error bars indicate sem. Bars with the same letter (a to d) in the same graph did not differ significantly (p > 0.05).

To determine the *XYLA*-dependent ability of *C. sonorensis* transformants to produce L-lactic acid anaerobically several strains were cultivated in flasks sealed with water locks in CaCO_3_buffered YP medium with 50 g l^−1^ D-xylose. All *XYLA* containing strains consumed significantly (p < 0.05) more D-xylose than C29, although D-xylose consumption by most strains was low (Figure 3).

D-xylose consumption generally reflected the ability of the strains to produce L-lactic acid. Strain C311 (*xyl1Δ::XYLA*) appeared to produce slightly more L-lactic acid (1.8 ± 0.4 g l^−1^) than C29 (0.9 ± 0.1 g l^−1^), although the amounts produced by either strain were very low and did not differ significantly (p > 0.05, Figure 3). Additional expression of *ScXKS1* (*xyl1Δ::XYLA y::ScXKS1*, C341 and C342) resulted in a significant (p < 0.05) increase in L-lactic acid production (2.5 ± 0.3 g l^−1^) compared with C29 (Figure 3). Strains C345 and C346, from which both *XYL2* and *XYL1* had been deleted and in which both *XYLA* and *ScXKS1* were expressed, produced significantly (p < 0.05) more L-lactic acid (5.0 ± 0.3 g lactic acid l^−1^) than the other strains containing only *XYLA* (Figure 3).

L-lactic acid production was highest (p < 0.05) in C349 (8.5 ± 0.2 g l^−1^), in which two copies of *XYLA* were expressed in a *xyl1Δ, xyl2Δ, ScXKS1* background. However, the expression of two copies of *XYLA* in a *xyl1Δ* background (C313) or in a *ScXKS1 xyl1Δ* background (C348) in the presence of *XYL2* did not enhance L-lactic acid production. C313 and C348 produced similar (p > 0.05) amounts of L-lactic acid to strains C311, C341 and C342 (Figure 3).

Xylitol was produced only by C29 (0.9 ± 0.1 g l^−1^), which had the endogenous D-xylose utilisation pathway, and by strains C311 (0.9 ± 0.3 g l^−1^) and C313 (1.0 ± 0.4 g l^−1^), containing one or two copies of the xylose isomerase encoding gene, an intact *XYL2* but no *XYL1*.

## Discussion

The expression of the *ldhL* gene in *C. sonorensis* resulted in transformants that produced L-lactic acid from D-xylose. The maximum L-lactic acid concentration in CaCO_3_-buffered minimal medium cultivations was 27 g l^−1^ for strains with one copy of *ldhL* (C29, *PDC +* and C184, *PDC-*), regardless of whether *PDC* genes were present, and 31 g l^−1^ for the strain containing two copies of *ldhL* (C169, *PDC-*). Thus increasing the copy number of *ldhL* from one to two had only a small effect on L-lactic acid production while deletion of the *PDC* genes did not significantly (p > 0.05) affect L-lactic acid production (Table 1). C29, with one copy of the *ldhL* gene, did not produce acetate or ethanol during the cultivation even though the wild type did, showing that LDH was able to compete effectively with PDC for pyruvate in a microaerobic CaCO_3_-buffered D-xylose cultivation. The same phenomenon was observed on D-glucose medium with the L-lactate producing *C. sonorensis* strains with intact *PDC* genes [6].

In non-buffered conditions, strains with one (C184) and two (C169) copies of *ldhL* produced similar amounts of L-lactic acid, since production of only 13 g l^−1^ L-lactic acid had lowered the pH to 2.8, causing further uptake of D-xylose to stop. This was comparable with the 15 g l^−1^ L-lactic acid produced by *S. stipitis* [11] in non-buffered conditions in ~80 h. Both the production rate (0.31 g l^−1^ h^−1^) and yield of L-lactic acid on D-xylose (~0.54 g g^−1^) were higher than those previously published for production of L-lactic acid from D-xylose by yeast in non-buffered conditions [11].

Adaptation of cells to D-xylose strongly affected L-lactic acid production in cultivations with mixed sugars (D-glucose and D-xylose). The D-xylose consumption rate and L-lactic acid concentration were significantly (p < 0.05) higher and xylitol concentration was lower in cultures inoculated with D-xylose-grown cells, compared to cultures inoculated with D-glucose-grown cells (Figure 1). The enhanced L-lactic acid production with D-xylose-grown inoculum is probably due to higher xylose reductase, xylitol dehydrogenase and xylulokinase activities, which in turn reflected higher levels of transcription of the corresponding genes. Yeast cultures inoculated with D-xylose-grown cells had 2-, 16- and 3-fold higher xylose reductase, xylitol dehydrogenase and xylulokinase *in vitro* activities, respectively, compared to the cultures inoculated with D-glucose-grown cells (Figure 1), while the mRNA levels of *XYL1, XYL2* and *XKS1* genes were 3-, 5-, and 3-fold higher, respectively (Figure 2). The yeast *C. tenuis* [23], *S. stipitis* and *P. tannophilus* [24] also show enhanced xylose reductase and xylitol dehydrogenase activities in D-xylose-grown cells compared to D-glucose-grown cells, whereas there was no clear effect of the carbon source in *C. utilis* [12]. The effect and extent of this effect of carbon source on the activity of enzymes involved in D-xylose metabolism is apparently species dependent.

Fungal L-lactic acid production from D-xylose has been described in *S. stipitis* and *C. utilis* strains expressing the *LDH* gene of *L. helveticus* and *B. taurus*, respectively [11,12], and additionally in the filamentous fungus *R. oryzae* [8-10]. In the present study, the *C. sonorensis* transformant with one copy of *ldhL* (C29) produced amounts of L-lactic acid comparable to that produced by *S. stipitis* with one copy of *ldhL* [11] in CaCO_3_ buffered YNB medium with D-xylose, but at a lower rate (Table 5). The *C. sonorensis ldhL* strain utilised D-xylose more efficiently and produced more L-lactic acid with a higher yield and rate than *R. oryzae* CBS 112.07 in minimal D-xylose medium [10] or *C. utilis LDH* with only its endogenous D-xylose metabolic pathway [12] (Table 5). Over-expression of exogenous XR, XDH and XK encoding genes in *C. utilis* has, however, resulted in much higher L-lactic acid production than with the native pathway, particularly when a xylose reductase with dual cofactor specificity was introduced [12] (up to 67.2 g l^−1^, Table 5). Thus, both enzyme activity and co-factor availability may be limiting, even in D-xylose utilising strains.

**Table 5:** **L – lactic acid production from xylose with various yeast strains**

**Strain**	**Xylose (g l** ^**−1**^ **)**	**Cultivation time (h)**	**Lactic acid (g l** ^**−1**^ **)**	**Lactic acid production rate (g h** ^**−1**^ **l** ^**−1**^ **)**	**Lactic acid yield (g g** ^**−1**^ **)**	**Xylitol (g l** ^**−1**^ **)**	**Genetic modifications**	**Reference**
*Candida sonorensis*	50	168	26.7	0.16	0.53	0*	*Lactobacillus helveticus ldhL*	This study
*Rhizopus oryzae* CBS 147.22	59	190	15.2	0.08	0.38	2.0	Wild type	10
*Scheffersomyces stipitis*	50	72	31.0	0.43	0.60	≤ 1.3	*L. helveticus ldhL*	11
*S. stipitis*	100	147	58.0	0.39	0.58	≤ 1.3	*L. helveticus ldhL*	11
*Candida utilis*	100	75	2.9	0.04	0.03	1.2	*PDC-*, 2 x *Bos taurus ldhL*	12
*C. utilis*	100	75	43.8	0.58	0.44	25.4	*PDC-*, 2 x *B. taurus ldhL, Candida shehatae* XR (NADPH preferring), *C. shehatae* XDH (NAD^+^ dependent), *S. stipitis* XK	12
*C. utilis*	100	75	67.2	0.93	0.67	3.2	P*DC-,* 2 x *B. taurus ldhL, C. shehatae* XR (NADH preferring), *C. shehatae* XDH (NAD^+^ dependent), *S. stipitis* XK	12

L-Lactic acid production titre, rate and yield with various yeast or fungal strains in buffered minimal xylose medium. Initial xylose and xylitol amounts at the end of cultivations are also indicated. Genetic modifications (*ldhL* = L-lactate dehydrogenase, XR = xylose reductase, XDH = xylitol dehydrogenase, XK = xylulokinase) have been indicated. *All xylitol had been consumed at the end of cultivation, but *in maximum* 4.6 g l^−1^ xylitol had been produced.

In bacteria, D-xylose is converted to D-xylulose directly by xylose isomerase with no redox cofactors involved. A xylose isomerase gene has also been cloned from the obligatory anaerobic fungus *Piromyces* sp. E2 [25]. Its expression in *C. sonorensis* resulted in an active enzyme with *in vitro* activity of 100 mU mg^−1^ (Table 2), which was lower than what has been reported in *S. cerevisiae* (300 – 1100 mU mg^−1^, [19]), but comparable to the activity of the *E. coli* xylose isomerase in *Hansenula polymorpha* (50 – 190 mU mg^−1^, [26]). Even though the *in vitro* xylose isomerase activity was low, it was functional *in vivo*, as indicated by production of L-lactic acid from D-xylose in strains which had the xylose isomerase encoding gene expressed and xylose reductase and xylitol dehydrogenase encoding genes deleted.

Under anaerobic conditions L-lactic acid production was significantly (p < 0.05) improved in strains expressing *XYLA* and in which the endogenous D-xylose pathway was disrupted, compared to the strain having the native D-xylose pathway (Figure 3). L-lactic acid production was further improved by expressing *ScXKS1* in the *XYLA* strains with the disrupted native pathway (Figure 3). Like ethanol production in *H. polymorpha* with xylose isomerase [26], the best L-lactic acid production was detected in strains from which both xylose reductase and xylitol dehydrogenase encoding genes had been deleted and which also expressed *ScXKS1* (Figure 3). A similar result has been observed for anaerobic ethanol production with *C. sonorensis* strains with no *ldhL* added, but having *XYLA* alone or together with *ScXKS1* expressed and the xylose reductase gene with or without the xylitol dehydrogenase gene deleted (K. Koivuranta, unpublished data). Xylose isomerase activity was probably limiting, since strains with two copies of the gene produced more L-lactic acid than strains with only one copy, when both *XYL1* and *XYL2* were lacking and *ScXKS1* was expressed.

## Conclusions

*C. sonorensis* expressing the *ldhL* gene of *L. helveticus* converted D-xylose to L-lactic acid via the endogenous D-xylose pathway, especially when the strains were adapted on D-xylose to increase their xylose reductase, xylitol dehydrogenase and xylulokinase activities.

We demonstrated for the first time L-lactic acid production from D-xylose under anaerobic conditions. To obtain anaerobic L-lactic acid production, it was necessary to replace the endogenous D-xylose pathway with a xylose isomerase encoding gene. Under anaerobic conditions, the best L-lactic acid production occurred with the strain deleted of endogenous xylose reductase and xylitol dehydrogenase encoding genes and expressing genes of *Piromyces sp.* xylose isomerase and *S. cerevisiae* xylulokinase.

Promising results in both micro- and anaerobic D-xylose cultivations with *C. sonorensis* strains for L-lactic acid production encourages further strain development using both evolutionary engineering (mutagenesis and selection) and metabolic engineering (e.g. expression of the non-oxidative pentose phosphate pathway) to improve lactate production.

## Methods

### Strains and plasmids

Microbial strains and plasmids used in the study are listed in Tables 6 and 7. Plasmids pCM29 and pVR103 were provided by Cargill (formerly NatureWorks LLC and Cargill Dow). *Escherichia coli* DH5α (Gibco BRL, Gaithersburg, MD, USA) was routinely used as a host for cloning and manipulation. *C. sonorensis* ATCC32109 (American Type Culture Collection) was used throughout the study as a control and was the parental strain for all transformants generated in this work.

**Table 6:** **Plasmids used in the study**

**Plasmid**	**Description**	**Reference or source**
pCM29	*ScP*_*PDC1*_ *-hph-ScT*_*GAL10*_ *-XYLA*	C. Miller, NatureWorks LLC
pVR103	*ScP*_*TEF*_ *-ScXKS1-ScT*_*GAL10*_	V. Rajgarhia, NatureWorks LLC
pMI271	*CsP*_*TDH1*_ *-hph-ScT*_*GAL10*_	[6]
pMI278	*CsP*_*GPD1*_ *-G418* ^R^ *-ScT*_*MEL5*_ *-CsPPGK1-BmLDH-ScT*_*GAL10*_	[6]
pMI281	*CsP*_*TDH1*_ *-BmLDH-ScT*_*GAL10*_	This study
pMI400	*ScP*_*PDC1*_ *-hph-ScT*_*GAL10*_ *-XYLA*	This study
pMI403	*CsP*_*TDH1*_ *-G418* ^*R*^ *-ScT*_*MEL5*_ *-CsP*_*PGK1*_ *-XYLA-ScT*_*GAL10*_	This study
pMI406	*ScXKS1-ScT*_*GAL10*_	This study
pMI409	*CsXYL2 5′-CsP*_*TDH1*_ *-BmLDH-ScT*_*GAL10*_	This study
pMI410	*CsXYL2 5′-CsP*_*TDH1*_ *-BmLDH-ScT*_*GAL10*_ *-CsXYL2 3′*	This study
pMI411	*CsXYL1 5′-CsP*_*TDH1*_ *-BmLDH-ScT*_*GAL10*_	This study
pMI412	*CsXYL1 5′-CsP*_*TDH1*_ *-BmLDH-ScT*_*GAL10*_ *-CsXYL1 3′*	This study
pMI417	*CsXYL1 5′-CsP*_*TDH1*_ *-G418* ^*R*^ *-ScT*_*MEL5*_ *-CsP*_*PGK1*_ *-XYLA-ScT*_*GAL10*_ *-CsXYL1 3′*	This study
pMI423	*CsP*_*PGK1*_ *-XYLA-ScT*_*GAL10*_ *-CsP*_*TDH1*_ *-hph-ScT*_*GAL10*_	This study
pMI424	*CsXYL1 5′-CsP*_*TDH1*_ *-G418* ^*R*^ *-ScT*_*MEL5*_ *-CsP*_*PGK1*_ *-ScXKS1-ScT*_*GAL10*_ *-CsXYL1 3′*	This study
pMI425	*CsXYL2 5′-CsP*_*TDH1*_ *-hph-ScT*_*MEL5*_ *-CsP*_*PGK1*_ *-ScXKS1-ScT*_*GAL10*_ *-CsXYL2 3′*	This study
pKK02	*CsXYL2 5′-CsP*_*TDH1*_ *-hph-ScT*_*MEL5*_ *-CsXYL2 3′*	This study
pKK03	*CsXYL1 5′-CsP*_*TDH1*_ *-G418* ^*R*^ *-ScT*_*MEL5*_ *-CsXYL1 3′*	This study

**Table 7:** ***Candida sonorensis*** **ATCC32109 derived strains constructed and studied in this work**

**Strains**	**Description**	**Reference or plasmid(s) used**
C29	*x::ldhL*	[6]
C169	*pdc1∆::ldhL pdc2∆::ldhL*	[6]
C184	*pdc1∆::ldhL pdc2∆*	[6]
C281	*x:*:*ldhL y*::*XYLA*	pMI403
C282, C283, C284	*x*::*ldhL y*::*XYLA z*::*ScXKS1*	pMI403, pMI425
C311	*x*::*ldhL xyl1*∆::*XYLA*	pMI417
C313	*x*::*ldhL xyl1*∆::*XYLA y*::*XYLA*	pMI417
C345, C346	*x*::*ldhL xyl1*∆::*XYLA xyl2*∆::*ScXKS1*	pMI417, pMI425
C349	*x*::*ldhL xyl1*∆::*XYLA y*::*XYLA xyl2*∆::*ScXKS1*	pMI417, pMI425
C341, C342	*x*::*ldhL xyl1*∆::*XYLA y*::*ScXKS1*	pMI417, pMI425
C348	*x*::*ldhL xyl1*∆::*XYLA y*::*XYLA z*::*ScXKS1*	pMI417, pMI425
C684, C685, C686	*xyl2*∆	pKK02
C689, C690	*xyl1*∆	pKK03

*x*:: and *y*:: indicate that the site of integration is not known.

### Media and culture conditions

Microaerobic (100 rpm) flask cultivations were carried out using two sequential pre-cultures to inoculate the production phase, as follows. Pre-culture 1: YP (yeast extract 10 g l^−1^ w/v, peptone 20 g l^−1^ w/v) with 50 g l^−1^ D-glucose (YPD) or 50 g l^−1^ D-xylose (YPX) was inoculated with a single colony from agar-solidified YPX or YPD and grown o/n with shaking at 250 rpm at 30°C. Pre-culture 2: 2 × 50 ml of YNB (yeast nitrogen base without amino acids, 6.7 g l^−1^ (Difco, Sparks, MD, USA)) with 0.5 M 2-[N-Morpholino]ethanesulfonic acid (MES) pH 5.5 and 50 g l^−1^ D-glucose (YGM) or 50 g l^−1^ D-xylose (YXM) in 250 ml flasks were inoculated from pre-culture 1 to OD_600_ of 0.1-0.2 and grown o/n with shaking at 250 rpm at 30°C. The OD_600_ of pre-culture 2 was typically around 11 at the time when cells were collected and residual sugar remained in the medium. Lactate production phase: cells from the second pre-culture flasks were collected by centrifugation and transferred to 50 ml of YNB medium with 50 g l^−1^ D-xylose to give an initial OD_600_ of 10 – 15, then incubated at 100 rpm in 250 ml flasks containing 2 g CaCO_3_ (final concentration 40 g l^−1^ CaCO_3_) (YXC). Alternatively, *C. sonorensis* C169 strain (*PDC-,* 2* *ldhL*), was collected by centrifugation and transferred into 50 ml of YNB medium with 30 g l^−1^ D-glucose and 30 g l^−1^ D-xylose and 40 g l^−1^ CaCO_3_ (YGXC).

Microaerobic cultures were incubated with shaking at 100 rpm at 30°C. 2 ml samples were removed daily. Culture supernatant samples were analysed by HPLC for sugars and metabolites. For measurement of xylose reductase activity, the biomass was generated in YGM or YXM.

For anaerobic flask cultivations of the *C. sonorensis* strain expressing *Piromyces sp XYLA*, pre-cultures were obtained by inoculating 50 ml of YPD medium with 10 mM MgCl_2_ in 250 ml flasks with cells grown on agar-solidified YPD, and incubating o/n at 250 rpm, 30°C. OD_600_ was measured and the amount of cells equivalent to OD_600_ 12 in 50 ml was collected by centrifugation and resuspended in 50 ml of YPX medium with 10 mM MgCl_2_ and transferred into a 100 ml flask containing 1.2 g CaCO_3_ (final concentration 24 g l^−1^ CaCO_3_). Flasks were sealed with water locks. The cultures were incubated at 30°C with 100 rpm shaking. Samples were collected after 146 hours cultivation.

For xylose isomerase activity measurements, cultures of 50 ml YPD medium with 10 mM MgCl_2_ in 250 ml flasks were inoculated with cells grown on agar-solidified YPD and incubated o/n at 250 rpm, 30°C. A 5 ml aliquot was removed for determination of xylose isomerase activity.

Agar-solidified medium (YPD or YPX) contained 20 g l^−1^ carbohydrate, rather than 50 g l^−1^.

### Northern analysis

RNA was isolated from C169 cells cultivated overnight in YGM or YXM medium using Trizol reagent (Invitrogen, Carlsbad, CA, USA), denatured with glyoxal prior to electrophoresis as described in Sambrook and Russell [27], blotted onto nylon membranes (Hybond-N, Amersham Biosciences, Little Chalfont, UK), and hybridized with [α-^32^P]dCTP (Amersham Biosciences) labelled probes as described previously [28]. The *XYL1, XYL2* and *XKS1* genes were used as probes, in addition to *S. cerevisiae ACT1* and *C. sonorensis PGK1* genes, which were used to standardise the mRNA levels in quantification.

### Enzymes, primers, and chemicals

Restriction enzymes, DNA-modifying enzymes, and other molecular reagents were obtained from New England Biolabs (Ipswich, MA, USA), Thermo Scientific (Rockford, IL, USA), Promega (Wisconsin, MA, USA), Agilent Technologies (Santa Clara, CA, USA), and Roche (Germany). All common chemicals were purchased from Sigma (USA). Primers for PCR and sequencing were synthesized by Sigma Genosys (Little Chalfont, UK).

### Cloning of *XYL1, XYL2* and *XKS1* genes

Xylose reductase (*XYL1*), xylitol dehydrogenase (*XYL2*) and xylulokinase (*XKS1*) homologues were cloned from a *C. sonorensis* genomic lambda library [6] by hybridization. *XYL1* and *XKS1* genes were isolated as PCR products generated using degenerate oligonucleotide primers XR1, XR5, XK3 and XK6, corresponding to consensus sequences of known *XYL1* and *XKS1* genes (Table 8). The *XYL2* gene was isolated by using the *P. stipitis XYL2* gene [29] as a probe.

**Table 8:** **Oligonucleotides used in the study**

**Oligo name**	**Sequence**	**Use**
133PirXI	ggacatgcatgcatttggggtacccaaggccttccgctctagaaaacaatggctaaggaatatttcccacaaattc	*Piromyces* Sp. *XYLA*
134PirXI	ccaatgcattggttcctgcagggaattcgacaacatcaaagtctgggttagtg	*Piromyces* Sp. *XYLA*
Sc135XKS1ATG	aaggccttgcggccgcctctagaaaacaatgttgtgttcagtaattcagagac	*Saccharomyces cerevisiae XKS1*
Sc135XKS1Bgl2	gaaaaggccttgttcaatggaaatttagcctcgcg	*Saccharomyces cerevisiae XKS1*
Cs141XR	actgtcgagctcgtttaaaccttcaccttaaattccccaattgag	*Candida sonorensis XYL1* 5′ region
Cs142XR	actgacgcgtcgactcttgtttgattgtgtgttgattgatc	*Candida sonorensis XYL1* 5′ region
Cs143XR	ggcccgcggccgctaagcagctagtataggcaagatgtag	*Candida sonorensis XYL1* 3′ region
Cs144XR	gggacgggcccaactgtaataatccgactttcaacg	*Candida sonorensis XYL1* 3′ region
Cs137XDH	actgtcgagctcgtttaaacacctattcgggagtcaatcaaccat	*Candida sonorensis XYL2* 5′ region
Cs138XDH	actgacgcgtcgacgtatgtataataaggtatgattctgg	*Candida sonorensis XYL2* 5′ region
Cs139XDH	ggcccgcggccgctaggctagttttctaaaattttggtg	*Candida sonorensis XYL2* 3′ region
Cs140XDH	gggacgggcccaagtatgagaaatattgatgatatag	*Candida sonorensis XYL2* 3′ region
XR1	gghtaymgwttdttygayggtgc	*XYL1* degenerative oligo
XR5	ccadkyccawggrtyrttraatct	*XYL1* degenerative oligo
XK3	tcrtanarrttcatnccrca	*XKS1* degenerative oligo
XK6	tcracycarcarytsaa	*XKS1* degenerative oligo

Cloning of the xylulokinase (*XKS1*) gene resulted in a fragment with 1.8 kb coding region, and 1.5 kb upstream and 0.5 kb downstream regions from the open reading frame. In Southern analysis only one xylulokinase encoding gene was detected. The highest overall amino acid sequence identity, in comparison with known fungal xylulokinase encoding genes, was 68%.

### Construction of *XYL1* expression and targeting vectors

Plasmid pMI317 was constructed for the expression of *S. stipitis XYL1* [30] under the control of the *C. sonorensis PGK1* promoter [6]. The *S. stipitis XYL1* gene, encoding a NAD(P)H dependent xylose reductase, was obtained from plasmid pUA103 [31]. The plasmid also contained the hygromycin resistance gene (*E. coli hph*) for the selection of transformants.

For replacement of the *C. sonorensis XYL1* locus, a vector with *XYL1* targeting sequences was constructed. The *C. sonorensis XYL1* 5′ region was PCR amplified with primers Cs141XR and Cs142XR (Table 8) from a genomic lambda library clone, CsXRlambda 4. The PCR product was cut with *Sac*I + *Sal*I, the 0.6 kb fragment was isolated from a gel and ligated to a 5 kb *Sac*I + *Sal*I fragment of pMI281. The resulting plasmid was named pMI411. Vector pMI281 was prepared by cutting pMI278 [6] with *Xba*I, isolating and circularizing the 5 kb fragment. The *C. sonorensis XYL1* 3′ region was PCR amplified using primers Cs143XR and Cs144XR (Table 8) from the library clone CsXRlambda 4. The PCR product was cut with *Not*I + *Apa*I, the 0.6 kb fragment was gel purified and ligated to the 5.4 kb *Not*I + *Apa*I fragment of pMI411. The resulting plasmid was named pMI412.

### Construction of a *XYL2* targeting vector

For replacement of the *C. sonorensis XYL2* locus, a vector with *XYL2* targeting sequences was constructed. The *C. sonorensis XYL2* 5′ region was PCR amplified using primers Cs137XDH and Cs138XDH (Table 8) with the lambda library clone XDH lambda 1/1/1 as a template. The PCR product was cut with *Sac*I + *Sal*I, the 0.6 kb fragment was isolated from gel and ligated to the 5 kb *Sac*I + *Sal*I fragment of pMI281 to generate plasmid pMI409. The *C. sonorensis XYL2* 3′ region was PCR amplified using primers Cs139XDH and Cs140XDH (Table 8) and lambda library clone CsXDH lambda 1/1/1 as a template. The PCR product was cut with *Not*I-*Apa*I, the 0.5 kb fragment was isolated from a gel and ligated to the 5.6 kb *Not*I-*Apa*I fragment of pMI409 to generate plasmid pMI410.

### Construction of a *XYLA* expression vector

The vector containing the *Piromyces XYLA* gene [25] under the *C. sonorensis PGK1* promoter and *S. cerevisiae GAL10* terminator was constructed as follows. The *Piromyces XYLA* gene was modified by adding a *Kpn*I restriction site to the 5′ end of gene. First, the *Piromyces XYLA* region from the ATG start codon to a single *Age*I site was PCR amplified using primers 133PirXI and 134PirXI (Table 8) with pCM29 as template. The PCR product was cut with *Age*I-*Kpn*I and the 0.5 kb fragment was isolated from a gel. pCM29 was cut with *Age*I and the 8.2 kb fragment was gel purified, partially digested with *Kpn*I, and the 6.5 kb *Age*I-*Kpn*I fragment was gel purified and ligated to the 0.5 kb PCR product, generating plasmid pMI400. In the following step the *XYLA* gene was placed under control of the *C. sonorensis PGK1* promoter in a vector containing the G418^R^ marker gene. pMI278 was cut with *Bam*HI, filled in with Klenow enzyme and partially digested with *Xba*I. The 6.7 kb fragment was isolated from a gel. pMI400 was cut with *Sbf*I, made blunt ended with T4 polymerase, and cut with *Xba*I. The 1.3 kb fragment was isolated from a gel and ligated to the 6.7 kb fragment of pMI278. The resulting plasmid was named pMI403. In addition, *C. sonorensis XYL1* targeting sequences from pMI412 (see above), were incorporated into pMI403. pMI412 was cut with *Sal*I-*Not*I and the 4.0 kb fragment was isolated from a gel and ligated to the 5.0 kb *Sal*I-*Not*I fragment of pMI403. The resulting plasmid was named pMI417. Yeast transformations were carried out with *Sac*I-*Apa*I or *Pme*I- *Psp*OMI cut pMI417.

### Construction of *XKS1* expression vectors

The vector containing the *S. cerevisiae XKS1* gene [32] under the *C. sonorensis PGK1* promoter and *S. cerevisiae GAL10* terminator was constructed as follows. First, the G418 resistance gene in pMI403 was replaced with the hygromycin resistance gene. pMI403 was cut with *Eco*NI, filled in with Klenow enzyme, cut with *Sal*I and the 6.5 kb fragment was isolated from a gel. The hygromycin resistance gene was obtained from pMI271 [6] digested with *Bam*HI, filled in with Klenow enzyme, and digested with *Sal*I. The 1.7 kb *Bam*HI(blunt)-*Sal*I fragment was isolated from a gel and ligated to the 6.5 kb *Eco*NI(blunt)-*Sal*I fragment of pMI403. The resulting plasmid was named pMI423.

The 5′ end of *S. cerevisiae XKS1* gene was modified by PCR using primers Sc135XKS1ATG and Sc135XKS1Bgl2 (Table 8) with pVR103 as the template. The PCR product was cut with *Not*I-*Bgl*II, and the 0.3 kb fragment was ligated to the 4.6 kb *Not*I-*Bgl*II fragment of pVR103. The plasmid was named pMI406. Plasmid pMI425, containing the *S. cerevisiae XKS1* and the hygromycin resistance gene between *C. sonorensis XYL2* targeting sequences, was constructed by ligating together three fragments: a 5.0 kb *Xba*I-*Bam*HI fragment from pMI410, a 1.8 kb *Xba*I-*Bam*HI fragment from pMI406, and a 2.8 kb *Xba*I fragment from pMI423. Targeted integration (*XYL2* locus) in yeast transformations was carried out with *Pme*I-*Apa*I, *Sac*I-*Apa*I or *Pme*I-*Psp*OMI cut pMI425 and random integration was carried out with *Sal*I-*Not*I cut pMI425.

### Construction of *XYL1* and *XYL2* deletion cassettes

For the replacement of *C. sonorensis XYL2* with the hygromycin resistance gene, plasmid pMI425 was digested with *Afl*II and *Not*I, the ends were filled in with Klenow enzyme, and the 5.7 kb fragment was circularized to form pKK02. *Pme*I-*Psp*OMI cut pKK02 was used in yeast transformation.

For the deletion of the *C. sonorensis XYL1* gene, vector pMI424, with *XYL1* targeting sequences and the G418^R^ marker gene, was constructed by ligating three fragments, a 5.1 kb *Xba*I-*Bam*HI from pMI412, a 1.8 kb *Xba*I-*Bam*HI fragment from pMI406, and a 2.7 kb *Xba*I fragment from pMI278. The resulting plasmid was named pMI424. Plasmid pMI424 was cut with *Afl*II and *Not*I and filled in with Klenow enzyme, and the 5.6 kb fragment was circularized to form pKK03. *Pme*I-*Psp*OMI cut pKK03 was used in yeast transformation.

### Construction of *C. sonorensis* strains

Strains with one copy of *ldhL* (C29) and with *PDC1* and *PDC2* both deleted with one or two copies of *ldhL* (C184 and C169, respectively) were constructed previously [6]. *C. sonorensis* was transformed using the lithium acetate method [33]. Transformants constructed for the present work were selected on YPD-hygromycin plates (200 μg/ml) or YPD-G418^R^ plates (200 μg/ml), as appropriate. The *C. sonorensis* strains generated by transformation are listed in Table 7.

### Preparation of crude extracts and enzyme assays

Cell free extracts for enzyme activity measurements were prepared using Y-PER Yeast Protein Extraction Reagent (Thermo Scientific, Rockford, IL, USA). Protein concentrations were determined with the Advanced Protein Assay Reagent (Cytoskeleton Inc, Denver, CO, USA; for results in Table 3) or with the Lowry method [34]. Bovine serum albumin (Sigma, USA) was used as protein standard. Activities are expressed in units per milligram of protein. One U was defined as the amount of enzyme required to reduce 1 μmol of substrate per min. The XR, XDH and XI assays were performed in a Cobas Mira automated analyser (Roche, Germany).

Xylose reductase and xylitol dehydrogenase activities were determined as described previously [35,36]. Xylose isomerase activity was determined by monitoring the oxidation of NADH at 340 nm at +30°C. The reaction mixture contained 100 mM TES (2-[[1,3-dihydroxy-2-(hydroxymethyl)propan-2-yl]amino]ethanesulfonic acid)-NaOH, pH 7.0, 250 mM D-xylose, 10 mM MgCl_2_, 0.6 mM NADH and 2.5 U sorbitol dehydrogenase. Background was determined by measuring the activity without D-xylose.

Xylulokinase activity was determined in a two-step assay. In the first step the reaction mixture contained 50 mM HEPES ((4-(2-hydroxyethyl)-1-piperazineethanesulfonic acid)/KOH pH 7.5, 5 mM ATP, 6 mM MgCl_2_, 20 mM xylulose. The background reaction was determined by adding water instead of D-xylose. After adding the enzyme (sample) the reaction was incubated at +30°C for 0 and 240 seconds. The reaction was stopped by incubation at +95°C for 5 min. In the second step , 40 mM HEPES-KOH pH 7.5, 10 mM MgCl_2_, 2.5 mM phosphoenol pyruvate, 0.2 mM NADH were added to the reaction mixture and the absorbance at 340 nm was measured before adding a mixture of myokinase (10 Units per reaction), pyruvate kinase (3.5 Units per reaction) and lactate dehydrogenase (10 Units per reaction). The reaction mixture was incubated for 1 hour and absorbance at 340 nm was measured. The xylulokinase activity was calculated from the ADP produced during the reaction.

### Analytical methods

Culture supernatants were analysed by HPLC for L-lactic acid, D-xylose, xylitol, xylulose, pyruvic acid, acetic acid, glycerol and ethanol, as described in Ilmén et al. [6].

### Nucleotide sequence accession numbers

Sequence data from this article have been deposited with the EMBL database under the accession numbers HE792813, HE792814 and HE792815.

### Statistical analysis

Data is given as mean ± standard error of the mean (sem). Significant differences between two strains or conditions were determined by the Student t-test. Differences between three or more strains or conditions were assessed by analysis of variance (ANOVA) and Fisher's multiple range test, when appropriate.
